# Body Mass Index Protects Against Temporomandibular Disorders, Whereas Systemic Inflammation, Vitamin D, and Migraine Do Not: A Causal Inference Study

**DOI:** 10.1155/prm/1109763

**Published:** 2026-11-02

**Authors:** Miao Yu, Li Dai

**Affiliations:** ^1^ Department of Stomatology Shandong Provincial Hospital Affiliated to Shandong First Medical University Jinan 250000 China sph.com.cn

**Keywords:** body mass index, C-reactive protein, causal inference, Mendelian randomization, migraine, temporomandibular disorders (TMDs), vitamin D

## Abstract

**Background and Objectives:**

Observational studies link temporomandibular disorders (TMDs) with body mass index (BMI), vitamin D, C‐reactive protein (CRP), and migraine but causal relationships remain unclear. This study aimed to assess the bidirectional causal effects between these factors and TMD using Mendelian randomization (MR).

**Materials and Methods:**

We used publicly available GWAS summary statistics. TMD data were from FinnGen R13 (23,035 cases; 477,151 controls). Exposure data for BMI, vitamin D, CRP, and migraine were from UK Biobank, SUNLIGHT, CHARGE, and FinnGen. Inverse‐variance weighted (IVW) analysis was the primary method, with multiple sensitivity analyses and stratified MR.

**Results:**

Higher genetically predicted BMI significantly reduced TMD risk (IVW: OR = 0.84, 95% CI: 0.79–0.89, *p* = 6.9 × 10^−8^), with consistent dose–response gradients across BMI strata. No significant causal effects were found for vitamin D (OR = 1.00, *p* = 0.932), CRP (OR = 1.03, *p* = 0.502), or migraine (OR = 1.00, *p* = 0.653). Reverse MR found no causal effects of TMD on any factor.

**Conclusions:**

Higher BMI protects against TMD, whereas vitamin D, CRP, and migraine do not causally influence TMD risk. These findings challenge the inflammation‐deficiency hypothesis and highlight the role of body composition in TMD.

## 1. Introduction

Temporomandibular disorders (TMD) comprise a group of musculoskeletal conditions affecting the temporomandibular joint, masticatory muscles, and associated structures, with a global prevalence of approximately 5%–12% [1]. Clinical manifestations include orofacial pain, joint sounds, limited mouth opening, and impaired masticatory function, substantially impairing patients’ quality of life [2]. Despite this considerable disease burden, the etiology and pathogenesis of TMD remain incompletely understood [3].

Several systemic factors have been implicated in TMD development and progression. Elevated serum C‐reactive protein (CRP) levels have been reported in TMD patients, suggesting that systemic inflammation may contribute to TMD pathogenesis [4]. Vitamin D deficiency has also been associated with TMD, potentially through aberrant bone metabolism and impaired muscle function [5]. Furthermore, TMD and migraine exhibit high clinical comorbidity, sharing trigeminovascular sensitization pathways [6]. The relationship between body mass index (BMI) and TMD is particularly complex—some studies identify obesity as a risk factor, whereas others report that low BMI is associated with increased TMD risk [7].

Conventional observational studies, however, are susceptible to confounding and reverse causation, precluding causal inference. For example, TMD‐related chronic pain may lead to reduced physical activity and subsequent BMI changes, rather than BMI directly influencing TMD risk [8]. Mendelian randomization (MR) leverages genetic variants as instrumental variables (IVs) to overcome these limitations, attenuating confounding bias and reverse causation when estimating causal effects [9].

To date, MR studies examining these factors in relation to TMD remain extremely limited. To our knowledge, no published MR study has systematically evaluated the bidirectional causal relationships between CRP, vitamin D, migraine, BMI, and TMD. Therefore, the present study aimed to (1) assess the causal effects of these four exposures on TMD risk, (2) examine the reverse causal effects of TMD on these factors, and (3) verify the robustness of findings through multiple sensitivity analyses.

## 2. Methods

### 2.1. Study Design

This study employed a bidirectional two‐sample MR design. In the forward MR, CRP, 25(OH) vitamin D, migraine, and BMI served as exposures, with TMD as the outcome. In the reverse MR, TMD served as the exposure, with the four factors as outcomes. All analyses used publicly available GWAS summary statistics; therefore, no additional ethical approval was required.

### 2.2. Data Sources

TMD GWAS summary statistics were obtained from FinnGen Release 13 (R13), defined by ICD‐10 code K07 (diseases of temporomandibular joint), encompassing both inpatient and outpatient records. This dataset included 23,035 cases and 477,151 controls (total *N* = 500,186), comprising 21,327,043 single‐nucleotide polymorphisms (SNPs).

CRP GWAS data were from the CHARGE Consortium (GWAS ID: ebi‐a‐GCST90012053), including approximately 200,000 European‐ancestry participants. Serum 25(OH) vitamin D GWAS data were from the SUNLIGHT Consortium (GWAS ID: ebi‐a‐GCST005195), including approximately 80,000 European‐ancestry participants. Migraine GWAS data were from FinnGen R13 (phenotype code: G6_MIGRAINE), including 28,504 cases and 366,556 controls. BMI GWAS data were from UK Biobank via the IEU OpenGWAS platform (GWAS ID: ieu‐b‐40), including approximately 681,275 European‐ancestry participants. Detailed information on each GWAS is provided in Supporting Table S1.

### 2.3. IV Selection

For forward MR, SNPs reaching genome‐wide significance (*p* < 5 × 10^−8^) were extracted from each exposure GWAS as candidate IVs. If fewer than two SNPs met this threshold, the significance level was sequentially relaxed to 1 × 10^−6^ and 5 × 10^−6^. Linkage disequilibrium (LD) clumping was subsequently performed using a physical distance window of 1000 kb, retaining only the SNP with the smallest *p* value within each window. F‐statistics were calculated for each IV (*F* = *β* ^2^/SE^2^); *F* > 10 indicated the absence of weak instrument bias [10]. For reverse MR, TMD was the exposure with the same selection strategy. The top 20 IVs for BMI are listed in Supporting Table S2.

### 2.4. MR Analysis

The inverse‐variance weighted (IVW) method served as the primary analysis [11]. Complementary methods included MR–Egger regression, weighted median, weighted mode, and simple mode [12–14]. Effect estimates were expressed as odds ratios (ORs) with 95% confidence intervals (CIs) per unit increase in each exposure.

### 2.5. Sensitivity Analysis

Heterogeneity among IVs was assessed using Cochran’s Q test. Horizontal pleiotropy was evaluated through the MR–Egger regression intercept. Leave‐one‐out analysis was performed to assess the influence of individual SNPs on the overall estimates [15]. All statistical analyses were conducted in R Version 4.5.0 using the TwoSampleMR (Version 0.7.9) and ggplot2 packages. Statistical significance was set at *p* < 0.05 (two‐sided).

## 3. Results

### 3.1. IV Characteristics

In the forward MR analyses, the numbers of IVs and mean F‐statistics were as follows: CRP, 5 SNPs (*F* = 221); vitamin D, 60 SNPs (*F* = 67); migraine, 20 SNPs (*F* = 35); and BMI, 50 SNPs (*F* = 65) (Table 1). All mean F‐statistics substantially exceeded the conventional threshold of 10, confirming the absence of weak instrument bias. In the reverse MR analyses, TMD harbored only two genome‐wide significant SNPs (*F* = 32).

**TABLE 1:** Forward Mendelian randomization results.

Exposure	N SNPs	Mean F	IVW OR (95% CI)	IVW P	MR–Egger P	Weighted median P	Q P	Intercept P
CRP	5	221	1.03 (0.94–1.13)	0.502	0.318	0.677	0.153	0.113
Vitamin D	60	67	1.00 (0.96–1.04)	0.932	0.353	0.181	0.422	0.320
Migraine	20	35	1.00 (0.99–1.01)	0.653	0.219	0.977	—	—
BMI	**50**	**65**	**0.84 (0.79–0.89)**	**6.9 × 10** ^**−**^ **^8^**	**8.2 × 10** ^**−**^ **^6^**	**1.4 × 10** ^**−**^ **^8^**	—	—

*Note:* Q P, Cochran’s Q test *p* value; Intercept P, MR–Egger intercept test *p* value. The bold values indicate the results for the exposure (BMI) that reached statistical significance (*p* < 0.05 ) and represents the primary positive finding of this study.

Abbreviations: CI, confidence interval; IVW, inverse‐variance weighted; OR, odds ratio.

### 3.2. Forward MR: Effects of Exposures on TMD

BMI and TMD: Genetically predicted higher BMI was significantly associated with reduced TMD risk (IVW: OR = 0.84, 95% CI: 0.79–0.89, *p* = 6.9 × 10^−8^) (Figures 1 and 2). The weighted median (OR = 0.76, *p* = 1.4 × 10^−8^) and MR–Egger regression (OR = 0.67, *p* = 8.2 × 10^−6^) produced consistent directional estimates (Table 1). Leave‐one‐out analysis demonstrated that no SNP drove the overall effect (Figure 3). Funnel plot analysis revealed no evidence of directional pleiotropy (Figure 4). Cochran’s Q test revealed no significant heterogeneity, and the MR–Egger intercept test detected no evidence of horizontal pleiotropy. Full heterogeneity and pleiotropy test results are presented in Supporting Table S4. Stratified MR analysis by BMI effect size tertiles showed consistent protective effects across all strata: low BMI group (OR = 0.689, 95% CI: 0.634–0.750), mid BMI group (OR = 0.738, 95% CI: 0.690–0.789), and high BMI group (OR = 0.758, 95% CI: 0.711–0.808) (all *p* < 0.0001; Supporting Table S6). A modest attenuation of the protective effect was observed with increasing BMI effect size, suggesting a dose–response gradient rather than a U‐shaped relationship.

**FIGURE 1:**
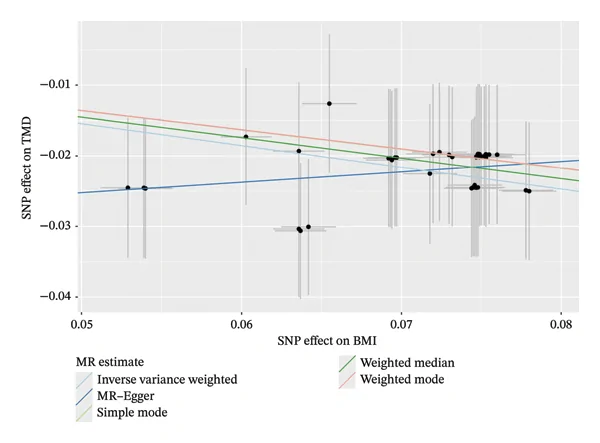
Scatter plot of SNP effects on body mass index (BMI) versus temporomandibular disorders (TMDs). Each point represents a SNP plotted by its effect on BMI (*x*‐axis, standard deviation units) and its effect on TMD (*y*‐axis, log odds ratio). Colored lines represent the slope estimated by different Mendelian randomization methods. The inverse‐variance weighted (IVW) method (light blue) served as the primary analysis.

**FIGURE 2:**
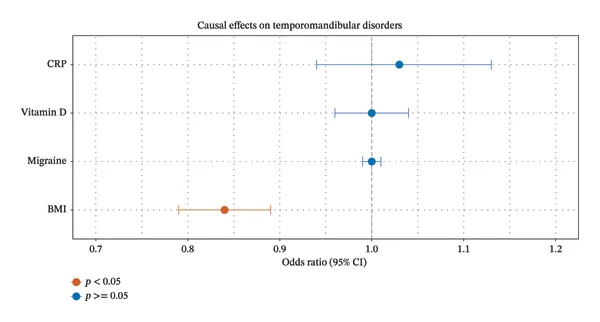
Forest plot summarizing the causal effects of all four exposures on TMD risk. Squares represent the IVW causal effect estimate (odds ratio) for each exposure, with horizontal lines indicating 95% confidence intervals. BMI (orange) showed a significant protective effect (OR = 0.84, 95% CI: 0.79–0.89, *p* = 6.9 × 10^−8^), whereas CRP, vitamin D, and migraine (blue) showed no significant causal associations with TMD.

**FIGURE 3:**
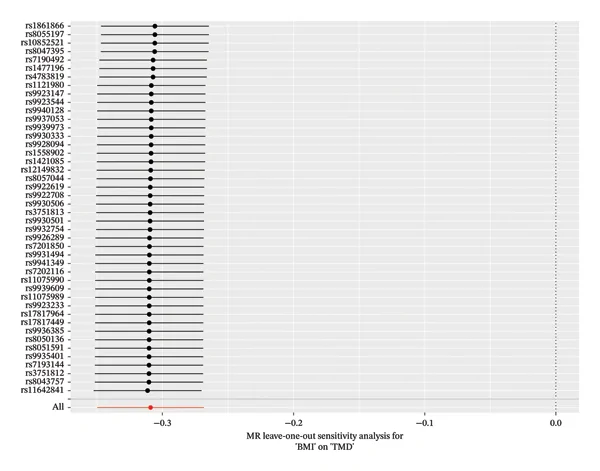
Leave‐one‐out analysis for the causal effect of BMI on TMD. Each row shows the IVW estimate after removing the corresponding SNP from the analysis. The red line represents the overall IVW estimate including all SNPs. Error bars indicate 95% confidence intervals. No single SNP substantially influenced the overall causal estimate.

**FIGURE 4:**
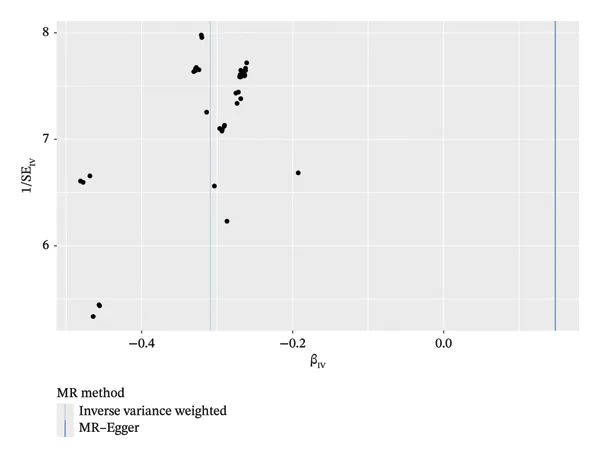
Funnel plot of individual SNP causal effect estimates for BMI on TMD. Each point represents a SNP plotted by its causal effect estimate (*x*‐axis) and the inverse of its standard error (*y*‐axis). The vertical line indicates the IVW causal estimate. Symmetry around the IVW estimate suggests the absence of directional pleiotropy.

CRP and TMD: No significant causal association was found between genetically predicted CRP levels and TMD risk (IVW: OR = 1.03, 95% CI: 0.94–1.13, *p* = 0.502) (Figure 2, Table 1). Sensitivity analyses yielded consistent results, with no evidence of heterogeneity (Cochran’s Q *p* = 0.153) or horizontal pleiotropy (MR–Egger intercept *p* = 0.113).

Vitamin D and TMD: No significant causal association was observed (IVW: OR = 1.00, 95% CI: 0.96–1.04, *p* = 0.932) (Figure 2, Table 1). Sensitivity analyses confirmed the robustness of this null finding (Cochran’s Q *p* = 0.422; MR–Egger intercept *p* = 0.320).

Migraine and TMD: No significant causal association was detected (IVW: OR = 1.00, 95% CI: 0.99–1.01, *p* = 0.653) (Figure 2, Table 1). All complementary MR methods produced consistent estimates.

Complete sensitivity analysis results for all forward MR analyses, including MR–Egger, weighted median, weighted mode, and simple mode estimates, are provided in Supporting Table S3.

### 3.3. Reverse MR: Effects of TMD on Exposures

For TMD⟶CRP, only one SNP was available after harmonization, revealing no significant effect (*p* = 0.921). For TMD ⟶ vitamin D and TMD ⟶ BMI, no SNPs remained after harmonization, precluding MR analysis. For TMD⟶migraine, no significant effect was observed (IVW: *p* = 0.974). Detailed reverse MR results are presented in Table 2 and Supporting Table S5.

**TABLE 2:** Reverse Mendelian randomization results.

Outcome	TMD IVs	Harmonized SNPs	IVW P	Conclusion
CRP	2	1	0.921	No causal effect
Vitamin D	2	0	—	No SNP available
Migraine	2	97,120	0.974	No causal effect
BMI	2	0	—	No SNP available

*Note:* IVs, instrumental variables.

## 4. Discussion

This bidirectional two‐sample MR study systematically evaluated the causal relationships between four systemic factors and TMD. The principal finding was that genetically predicted higher BMI exerts a protective causal effect on TMD (OR = 0.84, *p* = 6.9 × 10^−8^), whereas CRP, vitamin D, and migraine showed no significant causal effects. Reverse MR analyses revealed no causal effects of TMD on any of the four factors.

It is important to emphasize that MR provides genetic evidence for causal inference under specific assumptions (relevance, independence, and exclusion restriction), but it does not constitute standalone proof of causality. The findings should be interpreted alongside physiological, molecular, and clinical evidence. Genetic estimates reflect lifelong exposure effects and may not directly translate to the magnitude of effect achievable through clinical interventions.

### 4.1. Protective Effect of BMI on TMD

The finding that higher BMI attenuates TMD risk challenges the conventional view of obesity as a universal risk factor for musculoskeletal disorders. Observational studies on the BMI–TMD relationship have yielded conflicting results. While some report a positive association between obesity and TMD pain, others find that individuals with low BMI are more susceptible to TMD [7]. Our MR results support the latter, providing genetic‐level evidence for a protective effect of higher BMI on TMD.

Several hypothetical mechanisms could potentially account for this protective effect although direct evidence is lacking. First, higher BMI might reflect greater muscle mass, and masticatory muscle function could correlate with systemic muscle status; however, this remains speculative without direct measurements of masticatory muscle parameters. Second, adipose tissue–derived estrogen has been proposed to exert protective effects on articular cartilage [16], but whether this pathway is relevant to the temporomandibular joint is unknown. Third, individuals with low BMI may be more susceptible to psychological stress, a well‐established risk factor for TMD [17], although this pathway was not directly assessed in our study. These mechanisms should be considered hypothesis‐generating and warrant investigation in future studies incorporating direct physiological and molecular measurements.

Stratified MR analysis revealed a consistent dose–response pattern rather than a U‐shaped relationship, with protective effects persisting across all BMI tertiles. The modest attenuation of the protective effect in higher BMI strata may reflect a ceiling effect, beyond which additional BMI increases confer diminishing protection. Future studies with individual‐level data are warranted to fully characterize the shape of the BMI–TMD relationship.

### 4.2. Null Findings for CRP, Vitamin D, and Migraine

The absence of a causal effect of CRP on TMD contrasts sharply with observational studies reporting elevated CRP levels in TMD patients [4]. Previous observational studies have reported elevated CRP levels [4] and lower vitamin D levels in TMD patients, as well as high comorbidity between migraine and TMD [6]. Our null MR findings contrast with these observations, suggesting that the observed associations may be driven by confounding factors—such as psychological stress, which can simultaneously influence inflammatory markers, dietary habits, and pain perception—or by reverse causation, whereby TMD‐related pain leads to lifestyle changes that alter CRP or vitamin D levels. Alternatively, the cross‐sectional nature of most observational studies precludes temporal inference, and the possibility of publication bias favoring positive associations cannot be excluded. These discrepancies highlight the importance of triangulating evidence from different study designs.

Similarly, the null findings for vitamin D and migraine carry important implications. Despite widespread belief in the role of vitamin D in musculoskeletal health [5], our MR analysis does not support a causal effect on TMD. The high clinical comorbidity between migraine and TMD [6] likely reflects shared trigeminovascular sensitization pathways rather than a unidirectional causal relationship. These findings underscore the importance of distinguishing correlation from causation in TMD research.

### 4.3. Strengths and Limitations

To our knowledge, this is the first bidirectional MR study to simultaneously evaluate four systemic factors in relation to TMD. The use of large‐scale GWAS data (total N exceeding 1,000,000) provided adequate statistical power, and multiple sensitivity analyses confirmed result robustness. The bidirectional design facilitated differentiation of causal direction.

Several limitations warrant acknowledgment. First, all GWAS data were derived from European populations, which may limit generalizability to other ethnicities. Second, BMI and TMD data originated from different GWAS sources, precluding colocalization analysis due to limited SNP overlap. Third, the reverse MR analyses were underpowered owing to the paucity of TMD‐associated SNPs (*n* = 2), which reflects the relatively modest heritability of TMD. Fourth, MR assesses linear effects of genetically predicted exposures and cannot capture potential nonlinear relationships, such as a U‐shaped BMI–TMD association. Fifth, the LD clumping strategy relied on physical distance rather than a reference LD panel, which may be less precise. Sixth, potential sample overlap between FinnGen‐based migraine and TMD datasets could introduce bias although the large sample sizes mitigate this concern. Finally, future prospective multicenter studies involving diverse populations from different continents (e.g., North America, Africa, Asia, and Australia) are warranted to assess BMI, dietary patterns, CRP, and vitamin D levels in TMD patients. Such studies would help determine whether BMI influences TMD etiopathogenesis across different genetic and environmental contexts.

### 4.4. Clinical Implications

These findings hold potential implications for TMD clinical management. Routine serum CRP or vitamin D testing is not supported for TMD risk assessment or management. Clinicians should remain attentive to the BMI and nutritional status of TMD patients—those with low BMI may face elevated TMD risk, and appropriate nutritional support and muscle function training may prove beneficial for TMD prevention and treatment. Future research should explore the mediating pathways between BMI, masticatory muscle function, and TMD, ideally through prospective cohort studies incorporating direct measurements of body composition.

## 5. Conclusion

These findings are hypothesis‐generating and should not be interpreted as clinical directives. Routine serum CRP or vitamin D testing is not supported for TMD risk assessment or management. The relationship between BMI and TMD warrants further clinical investigation, particularly in prospective studies with direct measurements of body composition, masticatory muscle function, and nutritional status [18].

## Author Contributions

Miao Yu designed and performed the research; Li Dai provided conception analyzed the data and revised the manuscript.

## Funding

This study was supported by the Natural Science Foundation of Shandong Province (ZR2023QH485 and ZR2023QH328).

## Ethics Statement

This study used publicly available, deidentified summary‐level genome‐wide association study (GWAS) data from FinnGen, UK Biobank, CHARGE Consortium, and SUNLIGHT Consortium. All original studies obtained ethical approval and informed consent from their respective institutional review boards. No new data were collected for this study.

This study was reviewed and approved by the Institutional Review Board of Shandong provincial hospital affiliated to Shandong First medical school (Approval No.:2023‐407). The study was conducted in accordance with the Declaration of Helsinki.

## Conflicts of Interest

The authors declare no conflicts of interest.

## Supporting Information

Additional supporting information can be found online in the Supporting Information section.

## Supporting information


**Supporting Information 1** Supporting Table S1 showed the detailed information on GWAS data sources. Supporting Table S2 showed top 20 instrumental variables for BMI ⟶ TMD analysis (ranked by F‐statistic). Supporting Table S3 showed complete sensitivity analysis results for all forward MR analyses. Supporting Table S4 was the results of heterogeneity and pleiotropy tests for forward MR analyses. Supporting Table S5 showed reverse MR full results. Supporting Table S6 showed stratified Mendelian randomization analysis of BMI on TMD by effect size tertiles.

**Supporting Information 2** STROBE‐MR‐checklist‐fillable.

## Data Availability

All GWAS summary statistics used in this study are publicly available. TMD and migraine data were obtained from FinnGen Release 13 (https://www.finngen.fi/en/access_results). BMI data were obtained from the IEU OpenGWAS platform (GWAS ID: ieu‐b‐40; https://gwas.mrcieu.ac.uk). CRP data were obtained from the IEU OpenGWAS platform (GWAS ID: ebi‐a‐GCST90012053). Vitamin D data were obtained from the IEU OpenGWAS platform (GWAS ID: ebi‐a‐GCST005195). All data are freely available for download without restriction. Detailed accession identifiers are provided in Supporting Table S1.
