# Supplementary material for: Body Mass Index Protects Against Temporomandibular Disorders, Whereas Systemic Inflammation, Vitamin D, and Migraine Do Not: A Causal Inference Study
Source: Pain Res Manag. 2026 Nov 2;2026:1109763. doi: 10.1155/prm/1109763 (PMC13728335; doi:10.1155/prm/1109763)
Supplement: Supplementary file 1 [file PRM-2026-1109763-s001.docx]

**STROBE-MR checklist of recommended items to address in reports of Mendelian randomization studies**^1^ ^2^

| **Item No.** | **Section** | **Checklist item** | **Page No.** | **Relevant text from manuscript** |
| --- | --- | --- | --- | --- |
| 1 | **TITLE and ABSTRACT** | Indicate Mendelian randomization (MR) as the study’s design in the title and/or the abstract if that is a main purpose of the study | 1 | Body Mass Index Protects Against Temporomandibular Disorders, Whereas Systemic Inflammation, Vitamin D, and Migraine Do Not: A Causal Inference Study |
|  | **INTRODUCTION** |  |  |  |
| 2 | **Background** | Explain the scientific background and rationale for the reported study. What is the exposure? Is a potential causal relationship between exposure and outcome plausible? Justify why MR is a helpful method to address the study question | 3 | 1)Scientific background and rationale :Temporomandibular disorders (TMD) comprise…of TMD remain incompletely understood.2)Exposure: Several systemic factors have been implicated in TMD development ... The relationship between body mass index (BMI) and TMD is particularly complex.3) Is a causal relationship plausible? "For example, TMD-related chronic pain may lead to reduced physical activity and subsequent BMI changes, rather than BMI directly influencing TMD risk."4) Why is MR helpful? "Mendelian randomization (MR) leverages genetic variants as instrumental variables to overcome these limitations, attenuating confounding bias and reverse causation when estimating causal effects." |
| 3 | **Objectives** | State specific objectives clearly, including pre-specified causal hypotheses (if any). State that MR is a method that, under specific assumptions, intends to estimate causal effects | 3 | 1)Specific objectives: "The present study aimed to: (1) assess the causal effects…multiple sensitivity analyses."2) MR estimates causal effects under assumptions: "Mendelian randomization (MR) leverages genetic variants as…estimating causal effects." |
|  | **METHODS** |  |  |  |
| 4 | **Study design and data sources** | Present key elements of the study design early in the article. Consider including a table listing sources of data for all phases of the study. For each data source contributing to the analysis, describe the following: |  |  |
|  | a) | Setting: Describe the study design and the underlying population, if possible. Describe the setting, locations, and relevant dates, including periods of recruitment, exposure, follow-up, and data collection, when available. | 4 | "This study employed a bidirectional two-sample MR design. In the forward MR analyses, CRP, 25(OH) vitamin D…factors as outcomes." |
|  | b) | Participants: Give the eligibility criteria, and the sources and methods of selection of participants. Report the sample size, and whether any power or sample size calculations were carried out prior to the main analysis | 4 | "This dataset comprised 23,035 …European-ancestry participants." |
|  | c) | Describe measurement, quality control and selection of genetic variants | 4 | "SNPs attaining genome-wide significance… absence of weak instrument bias." |
|  | d) | For each exposure, outcome, and other relevant variables, describe methods of assessment and diagnostic criteria for diseases | 4 | "TMD... defined by ICD-10 code K07 (diseases of temporomandibular joint), encompassing both inpatient and outpatient records." |
|  | e) | Provide details of ethics committee approval and participant informed consent, if relevant | 4 | "All analyses utilized publicly available GWAS summary statistics; therefore, no additional ethical approval was required." |
| 5 | **Assumptions** | Explicitly state the three core IV assumptions for the main analysis (relevance, independence and exclusion restriction) as well assumptions for any additional or sensitivity analysis | 4 | "F-statistics were calculated for each IV (F = β²/SE²)…conventional threshold of 10." "Horizontal pleiotropy was evaluated through the MR-Egger regression intercept." "Heterogeneity among IVs was assessed using … overall estimates." |
| 6 | **Statistical methods: main analysis** | Describe statistical methods and statistics used | 5 |  |
|  | a) | Describe how quantitative variables were handled in the analyses (i.e., scale, units, model) | 5 | Effect estimates were expressed as odds ratios (OR) with 95% confidence intervals (CI) per unit increase in each exposure |
|  | b) | Describe how genetic variants were handled in the analyses and, if applicable, how their weights were selected | 5 | SNPs attaining genome-wide significance (P < 5 × 10⁻⁸) were extracted... LD clumping... retaining only the SNP with the smallest P-value within each window. |
|  | c) | Describe the MR estimator (e.g. two-stage least squares, Wald ratio) and related statistics. Detail the included covariates and, in case of two-sample MR, whether the same covariate set was used for adjustment in the two samples | 5 | The inverse-variance weighted (IVW) method served as the primary analysis. Complementary methods included MR-Egger regression, weighted median, weighted mode, and simple mode. |
|  | d) | Explain how missing data were addressed | 5 | If fewer than two SNPs satisfied this threshold, the significance level was sequentially relaxed to 1 × 10⁻⁶ and 5 × 10⁻⁶. |
|  | e) | If applicable, indicate how multiple testing was addressed | 5 | Statistical significance was set at P < 0.05 (two-sided). |
| 7 | **Assessment of assumptions** | Describe any methods or prior knowledge used to assess the assumptions or justify their validity | 4,5 | Heterogeneity among IVs was assessed using Cochran's Q test… individual SNPs on the overall estimates. |
| 8 | **Sensitivity analyses and additional analyses** | Describe any sensitivity analyses or additional analyses performed (e.g. comparison of effect estimates from different approaches, independent replication, bias analytic techniques, validation of instruments, simulations) | 4,5 | Complementary methods included MR-Egger regression, weighted median, weighted mode, and simple mode... Reverse MR analyses... Stratified MR analysis by BMI effect size tertiles. |
| 9 | **Software and pre-registration** |  |  |  |
|  | a) | Name statistical software and package(s), including version and settings used | 5 | All statistical analyses were conducted in R version 4.5.0 using the TwoSampleMR (version 0.7.9) and ggplot2 packages. |
|  | b) | State whether the study protocol and details were pre-registered (as well as when and where) |  | Not pre-registered. |
|  | **RESULTS** |  |  |  |
| 10 | **Descriptive data** |  |  |  |
|  | a) | Report the numbers of individuals at each stage of included studies and reasons for exclusion. Consider use of a flow diagram | 5 | CRP: 5 SNPs (F = 221); vitamin D: 60 SNPs (F = 67); migraine: 20 SNPs (F = 35); and BMI: 50 SNPs (F = 65) |
|  | b) | Report summary statistics for phenotypic exposure(s), outcome(s), and other relevant variables (e.g. means, SDs, proportions) | 5 | All mean F-statistics substantially exceeded the conventional threshold of 10, confirming the absence of weak instrument bias. |
|  | c) | If the data sources include meta-analyses of previous studies, provide the assessments of heterogeneity across these studies | 5 | All GWAS data were derived from European populations. |
|  | d) | For two-sample MR:  i.  Provide justification of the similarity of the genetic variant-exposure associations between the exposure and outcome samples  ii.  Provide information on the number of individuals who overlap between the exposure and outcome studies | 6 | Potential sample overlap between FinnGen-based migraine and TMD datasets could introduce bias, although the large sample sizes mitigate this concern. |
| 11 | **Main results** |  |  |  |
|  | a) | Report the associations between genetic variant and exposure, and between genetic variant and outcome, preferably on an interpretable scale | 6 | Scatter plot of SNP effects on BMI versus TMD (Figure 1). |
|  | b) | Report MR estimates of the relationship between exposure and outcome, and the measures of uncertainty from the MR analysis, on an interpretable scale, such as odds ratio or relative risk per SD difference | 6 | Genetically predicted higher …TMD risk (IVW: OR = 1.03, P = 0.502)... No significant causal association was observed for vitamin D (IVW: OR = 1.00, P = 0.932)... No significant causal association was detected for migraine (IVW: OR = 1.00, P = 0.653). |
|  | c) | If relevant, consider translating estimates of relative risk into absolute risk for a meaningful time period |  |  |
|  | d) | Consider plots to visualize results (e.g. forest plot, scatterplot of associations between genetic variants and outcome versus between genetic variants and exposure) | 6 | Figure 1. Scatter plot... Figure 2. Leave-one-out analysis... Figure 3. Funnel plot... Figure 4. Forest plot. |
| 12 | **Assessment of assumptions** |  |  |  |
|  | a) | Report the assessment of the validity of the assumptions | 6 | Cochran's Q test revealed no significant heterogeneity, and the MR-Egger intercept test detected no evidence of horizontal pleiotropy. |
|  | b) | Report any additional statistics (e.g., assessments of heterogeneity across genetic variants, such as *I^2^*, Q statistic or E-value) | 6 | Cochran's Q P = 0.153 (CRP), P = 0.422 (Vitamin D); MR-Egger intercept P = 0.113 (CRP), P = 0.320 (Vitamin D). |
| 13 | **Sensitivity analyses and additional analyses** |  |  |  |
|  | a) | Report any sensitivity analyses to assess the robustness of the main results to violations of the assumptions | 6 | The weighted median (OR = 0.76, P = 1.4 × 10⁻⁸) and MR-Egger regression (OR = 0.67, P = 8.2 × 10⁻⁶) produced consistent directional estimates. |
|  | b) | Report results from other sensitivity analyses or additional analyses | 6 | Stratified MR analysis by BMI effect size … and high BMI group (OR = 0.758) (all P < 0.0001; Supplementary Table S6). |
|  | c) | Report any assessment of direction of causal relationship (e.g., bidirectional MR) | 6 | For TMD→CRP, only one SNP was available after harmonization…no significant effect was observed (IVW: P = 0.974). |
|  | d) | When relevant, report and compare with estimates from non-MR analyses |  |  |
|  | e) | Consider additional plots to visualize results (e.g., leave-one-out analyses) | 6 | Leave-one-out analysis demonstrated that no single SNP drove the overall effect (Figure 2). Funnel plot analysis revealed no evidence of directional pleiotropy (Figure 3). |
|  | **DISCUSSION** |  |  |  |
| 14 | **Key results** | Summarize key results with reference to study objectives | 9 | The principal finding was that genetically predicted higher BMI exerts a protective causal effect on TMD (OR = 0.84, P = 6.9 × 10⁻⁸), whereas CRP, vitamin D, and migraine showed no significant causal effects. Reverse MR analyses revealed no causal effects of TMD on any of the four factors |
| 15 | **Limitations** | Discuss limitations of the study, taking into account the validity of the IV assumptions, other sources of potential bias, and imprecision. Discuss both direction and magnitude of any potential bias and any efforts to address them | 8 | 1) Validity of assumptions :The reverse MR analyses were underpowered … reference LD panel, which may be less precise.2) Other sources of bias: All GWAS data were derived from European populations… migraine and TMD datasets. |
| 16 | **Interpretation** |  |  |  |
|  | a) | Meaning: Give a cautious overall interpretation of results in the context of their limitations and in comparison with other studies | 8 | The finding that higher BMI attenuates TMD risk challenges the conventional view of obesity as a universal risk factor for musculoskeletal disorders. |
|  | b) | Mechanism: Discuss underlying biological mechanisms that could drive a potential causal relationship between the investigated exposure and the outcome, and whether the gene-environment equivalence assumption is reasonable. Use causal language carefully, clarifying that IV estimates may provide causal effects only under certain assumptions | 8 | Higher BMI may reflect greater muscle mass... adipose tissue-derived estrogen exerts protective effects on articular cartilage... individuals with low BMI may be more susceptible to psychological stress. |
|  | c) | Clinical relevance: Discuss whether the results have clinical or public policy relevance, and to what extent they inform effect sizes of possible interventions | 8 | Routine serum CRP or vitamin D… support and muscle function training may prove beneficial. |
| 17 | **Generalizability** | Discuss the generalizability of the study results (a) to other populations, (b) across other exposure periods/timings, and (c) across other levels of exposure | 8 | All GWAS data were derived from European populations, which may limit generalizability to other ethnicities. MR assesses linear effects of genetically predicted exposures and cannot capture potential non-linear relationships. Stratified MR analysis revealed a consistent dose-response pattern rather than a U-shaped relationship, with protective effects persisting across all BMI tertiles. |
|  | **OTHER INFORMATION** |  |  |  |
| 18 | **Funding** | Describe sources of funding and the role of funders in the present study and, if applicable, sources of funding for the databases and original study or studies on which the present study is based | 9 | Supported by Natural Science Foundation of Shandong Province (ZR2023QH485 and ZR2023QH328) |
| 19 | **Data and data sharing** | Provide the data used to perform all analyses or report where and how the data can be accessed, and reference these sources in the article. Provide the statistical code needed to reproduce the results in the article, or report whether the code is publicly accessible and if so, where | 9 | All GWAS summary statistics …provided in Supplementary Table S1. |
| 20 | **Conflicts of Interest** | All authors should declare all potential conflicts of interest | 9 | The authors declare that they do not have any affiliation with or financial relationship/interest in a commercial organization that could pose a conflict of interest. |

This checklist is copyrighted by the Equator Network under the Creative Commons Attribution 3.0 Unported (CC BY 3.0) license.

1.	Skrivankova VW, Richmond RC, Woolf BAR, Yarmolinsky J, Davies NM, Swanson SA, et al. Strengthening the Reporting of Observational Studies in Epidemiology using Mendelian Randomization (STROBE-MR) Statement. JAMA. 2021;under review.

2.	Skrivankova VW, Richmond RC, Woolf BAR, Davies NM, Swanson SA, VanderWeele TJ, et al. Strengthening the Reporting of Observational Studies in Epidemiology using Mendelian Randomisation (STROBE-MR): Explanation and Elaboration. BMJ. 2021;375:n2233.
