# Supplementary material for: Body Mass Index Protects Against Temporomandibular Disorders, Whereas Systemic Inflammation, Vitamin D, and Migraine Do Not: A Causal Inference Study
Source: Pain Res Manag. 2026 Nov 2;2026:1109763. doi: 10.1155/prm/1109763 (PMC13728335; doi:10.1155/prm/1109763)
Supplement: Supplementary file 2 [file PRM-2026-1109763-s002.docx]

**Supplementary Table S1. Detailed information on GWAS data sources.**

| Trait | GWAS ID / Source | Population | Sample Size | Cases | Controls | SNPs |
| --- | --- | --- | --- | --- | --- | --- |
| TMD | FinnGen R13 | Finnish | 500,186 | 23,035 | 477,151 | 21,327,043 |
| CRP | ebi-a-GCST90012053 | European | ~200,000 | — | — | 13,089,174 |
| Vitamin D | ebi-a-GCST005195 | European | ~80,000 | — | — | 7,911,794 |
| Migraine | FinnGen R13 (G6_MIGRAINE) | Finnish | 395,060 | 28,504 | 366,556 | 21,325,312 |
| BMI | ieu-b-40 | European | 681,275 | — | — | 2,336,260 |

TMD, temporomandibular disorders; CRP, C-reactive protein; BMI, body mass index; SNP, single nucleotide polymorphism.

**Supplementary Table S2.** Top 20 instrumental variables for BMI → TMD analysis (ranked by F-statistic).

| Rank | SNP | Chr | Position | Effect Allele | Other Allele | Beta (BMI) | SE | P value | F |
| --- | --- | --- | --- | --- | --- | --- | --- | --- | --- |
| 1 | rs17817449 | 16 | 53813367 | G | T | 0.0750 | 0.0016 | <1×10⁻³⁰⁰ | 2197 |
| 2 | rs9935401 | 16 | 53816838 | A | G | 0.0749 | 0.0016 | <1×10⁻³⁰⁰ | 2191 |
| 3 | rs8050136 | 16 | 53816275 | A | C | 0.0748 | 0.0016 | <1×10⁻³⁰⁰ | 2186 |
| 4 | rs1558902 | 16 | 53803574 | A | T | 0.0780 | 0.0017 | <1×10⁻³⁰⁰ | 2105 |
| 5 | rs1421085 | 16 | 53800954 | C | T | 0.0778 | 0.0017 | <1×10⁻³⁰⁰ | 2094 |
| 6 | rs3751812 | 16 | 53818460 | T | G | 0.0755 | 0.0017 | <1×10⁻³⁰⁰ | 1972 |
| 7 | rs7193144 | 16 | 53810686 | C | T | 0.0753 | 0.0017 | <1×10⁻³⁰⁰ | 1962 |
| 8 | rs17817964 | 16 | 53828066 | T | C | 0.0753 | 0.0017 | <1×10⁻³⁰⁰ | 1962 |
| 9 | rs9936385 | 16 | 53819169 | C | T | 0.0752 | 0.0017 | <1×10⁻³⁰⁰ | 1957 |
| 10 | rs9939609 | 16 | 53820527 | A | T | 0.0749 | 0.0017 | <1×10⁻³⁰⁰ | 1941 |
| 11 | rs9930333 | 16 | 53799977 | G | T | 0.0748 | 0.0017 | <1×10⁻³⁰⁰ | 1936 |
| 12 | rs8051591 | 16 | 53816752 | G | A | 0.0748 | 0.0017 | <1×10⁻³⁰⁰ | 1936 |
| 13 | rs9937053 | 16 | 53799507 | A | G | 0.0747 | 0.0017 | <1×10⁻³⁰⁰ | 1931 |
| 14 | rs9923233 | 16 | 53819198 | C | G | 0.0747 | 0.0017 | <1×10⁻³⁰⁰ | 1931 |
| 15 | rs11075989 | 16 | 53819877 | T | C | 0.0747 | 0.0017 | <1×10⁻³⁰⁰ | 1931 |
| 16 | rs11075990 | 16 | 53819893 | G | A | 0.0747 | 0.0017 | <1×10⁻³⁰⁰ | 1931 |
| 17 | rs7202116 | 16 | 53821615 | G | A | 0.0747 | 0.0017 | <1×10⁻³⁰⁰ | 1931 |
| 18 | rs1417860482 | 16 | 53822651 | G | A | 0.0747 | 0.0017 | <1×10⁻³⁰⁰ | 1931 |
| 19 | rs9928094 | 16 | 53799905 | G | A | 0.0746 | 0.0017 | <1×10⁻³⁰⁰ | 1926 |
| 20 | rs9939973 | 16 | 53800568 | A | G | 0.0746 | 0.0017 | <1×10⁻³⁰⁰ | 1926 |

Chr, chromosome; SE, standard error; F, F-statistic.

*Note: All 20 SNPs are located within the FTO gene region on chromosome 16 (53.8 Mb), representing the strongest known BMI-associated locus. Complete list of all 50 independent instrumental variables after LD clumping is available upon request."*

**Supplementary Table S3. Complete sensitivity analysis results for all forward MR analyses.**

| Exposure | Method | N SNPs | Beta | SE | OR (95% CI) | P value |  |
| --- | --- | --- | --- | --- | --- | --- | --- |
| CRP | MR Egger | 5 | −0.070 | 0.059 | 0.93 (0.83–1.05) | 0.318 |  |
| CRP | Weighted median | 5 | −0.019 | 0.046 | 0.98 (0.90–1.07) | 0.677 |  |
| CRP | IVW | 5 | 0.032 | 0.048 | 1.03 (0.94–1.13) | 0.502 |  |
| CRP | Simple mode | 5 | 0.166 | 0.095 | 1.18 (0.98–1.42) | 0.155 |  |
| CRP | Weighted mode | 5 | −0.031 | 0.051 | 0.97 (0.88–1.07) | 0.576 |  |
|  |  |  |  |  |  |  |  |
| Vitamin D | MR Egger | 60 | −0.047 | 0.050 | 0.95 (0.86–1.05) | 0.353 |  |
| Vitamin D | Weighted median | 60 | −0.047 | 0.035 | 0.95 (0.89–1.02) | 0.181 |  |
| Vitamin D | IVW | 60 | −0.002 | 0.022 | 1.00 (0.96–1.04) | 0.932 |  |
| Vitamin D | Simple mode | 60 | −0.044 | 0.070 | 0.96 (0.83–1.10) | 0.534 |  |
| Vitamin D | Weighted mode | 60 | −0.052 | 0.046 | 0.95 (0.87–1.04) | 0.222 |  |
|  |  |  |  |  |  |  |  |
| Migraine | MR Egger | 12,968 | 0.123 | 0.100 | 1.13 (0.93–1.37) | 0.219 |  |
| Migraine | Weighted median | 12,968 | 0.0001 | 0.004 | 1.00 (0.99–1.01) | 0.977 |  |
| Migraine | IVW | 12,968 | 0.0014 | 0.003 | 1.00 (1.00–1.01) | 0.653 |  |
| Migraine | Simple mode | 12,968 | 0.115 | 0.211 | 1.12 (0.74–1.70) | 0.586 |  |
| Migraine | Weighted mode | 12,968 | 0.115 | 0.226 | 1.12 (0.72–1.75) | 0.611 |  |
|  |  |  |  |  |  |  |  |
| **BMI** | **MR Egger** | **652** | **−0.403** | **0.090** | **0.67 (0.56–0.80)** | **8.2×10⁻⁶** |  |
| **BMI** | **Weighted median** | **652** | **−0.269** | **0.047** | **0.76 (0.70–0.84)** | **1.4×10⁻⁸** |  |
| **BMI** | **IVW** | **652** | **−0.176** | **0.033** | **0.84 (0.79–0.89)** | **6.9×10⁻⁸** |  |
| **BMI** | **Simple mode** | **652** | **−0.232** | **0.197** | **0.79 (0.54–1.17)** | **0.240** |  |
| **BMI** | **Weighted mode** | **652** | **−0.398** | **0.110** | **0.67 (0.54–0.83)** | **3.3×10⁻⁴** |  |

IVW, inverse-variance weighted; OR, odds ratio; CI, confidence interval; SE, standard error.

**Supplementary Table S4. Heterogeneity and pleiotropy tests for forward MR analyses.**

| Exposure | Cochran's Q | Q P value | MR-Egger Intercept | Intercept SE | Intercept P value |
| --- | --- | --- | --- | --- | --- |
| CRP | 6.69 | 0.153 | 0.020 | 0.009 | 0.113 |
| Vitamin D | 60.48 | 0.422 | 0.004 | 0.004 | 0.320 |
| Migraine | — | — | — | — | — |
| BMI | — | — | — | — | — |

*Note: For migraine and BMI, heterogeneity and pleiotropy tests could not be computed due to computational limitations. However, consistency across multiple MR methods supports result robustness.*

**Supplementary Table S5. Reverse MR full results.**

| Exposure | Outcome | Method | N SNPs | Beta | SE | P value |
| --- | --- | --- | --- | --- | --- | --- |
| TMD | CRP | Wald ratio | 1 | 0.043 | 0.432 | 0.921 |
| TMD | Vitamin D | — | 0 | — | — | — |
| TMD | Migraine | MR Egger | 97,120 | −0.507 | 1.835 | 0.782 |
| TMD | Migraine | Weighted median | 97,120 | −0.001 | 0.001 | 0.220 |
| TMD | Migraine | IVW | 97,120 | −0.00002 | 0.001 | 0.974 |
| TMD | Migraine | Simple mode | 97,120 | 0.051 | 0.208 | 0.805 |
| TMD | Migraine | Weighted mode | 97,120 | 0.051 | 0.217 | 0.813 |
| TMD | BMI | — | 0 | — | — | — |

IVW, inverse-variance weighted; SE, standard error.

**Supplementary Table S6.** Stratified Mendelian randomization analysis of BMI on TMD by effect size tertiles.

| BMI Stratum | Beta Range | N SNPs | OR（95% CI） | P value |
| --- | --- | --- | --- | --- |
| Low BMI | 0.0529–0.0694 | 13 | 0.689 (0.634–0.750) | <0.0001 |
| Mid BMI | 0.0696–0.0747 | 15 | 0.738 (0.690–0.789) | <0.0001 |
| High BMI | 0.0747–0.0780 | 16 | 0.758 (0.711–0.808) | <0.0001 |
| **Overall** | **0.0529–0.0780** | **50** | **0.84 (0.79–0.89)** | **6.9 × 10⁻⁸** |
